# The impact of SLC10A3 on prognosis and immune microenvironment in colorectal adenocarcinoma

**DOI:** 10.1186/s40001-023-01526-4

**Published:** 2024-01-04

**Authors:** Bangting Wang, Wentao Fan, Yuwen Tao, Shijie Zhang, Jiankun Wang, Zhining Fan, Li Liu, Yan Wang

**Affiliations:** 1 Digestive Endoscopy Department, The First Affiliated Hospital With Nanjing Medical University and Jiangsu Province Hospital, Nanjing, Jiangsu China; 2 Gastroenterology Department, The Forth Affiliated Hospital With Nanjing Medical University, Nanjing, China; 3 The Friendship Hospital of Ili Kazkh Autonomous Prefecture, Ili & Jiangsu Joint Institute of Health, Yining, China

**Keywords:** SLC10A3, CRC, Biomarker, Immune infiltration, Prognosis

## Abstract

**Background:**

SLC10A3, a gene upregulated in pan-cancer, lacks full understanding regarding its prognostic implications and association with immune infiltration in colorectal cancer (CRC). This study comprehensively analyzed SLC10A3 in CRC, evaluating its prognostic significance and influence on the tumor's immune microenvironment.

**Methods:**

Transcriptomic data from TCGA were obtained to compare SLC10A3 expression in both colorectal cancer (CRC) and normal tissues. Prognostic value was assessed for overall survival (OS), disease-specific survival (DSS), and progression-free interval (PFI). DNA methylation patterns of SLC10A3 and correlation with DNA mismatch repair (MMR) were explored. Genetic alterations in SLC10A3 were scrutinized. The study also delved into the influence of SLC10A3 on the immune microenvironment of CRC, including immune cell infiltration and chemokines. Involvement of cancer-associated fibroblasts (CAFs) was explored. Methylation status of specific CpG islands in the SLC10A3 gene correlated with CRC patient prognosis. CRC tissue microarray was performed to verify the expression of SLC10A3 and its relationship with prognosis.

**Results:**

The research revealed that SLC10A3 is significantly upregulated in CRC and holds promise as a potential diagnostic marker. Elevated SLC10A3 expression was linked to poorer OS, DSS, and PFI. Methylation patterns of SLC10A3 displayed prognostic relevance, and genetic alterations in the gene were identified. SLC10A3 was shown to impact the immune microenvironment, with significant correlations observed between its expression and various immune cell types, chemokines, and markers associated with CAFs. Furthermore, an inverse relationship between SLC10A3 and MMR molecules was established. Methylation status of specific CpG islands within the SLC10A3 gene was associated with CRC patient prognosis. Tissue microarray showed that SLC10A3 was highly expressed in CRC and significantly correlated with poor prognosis.

**Conclusion:**

The study underscores the importance of elevated SLC10A3 in CRC, associating it with decreased survival and immune infiltration, proposing it as a diagnostic biomarker and appealing immunotherapy target, given its significant overexpression and influence on the immune microenvironment and prognosis through methylation patterns.

**Graphical Abstract:**

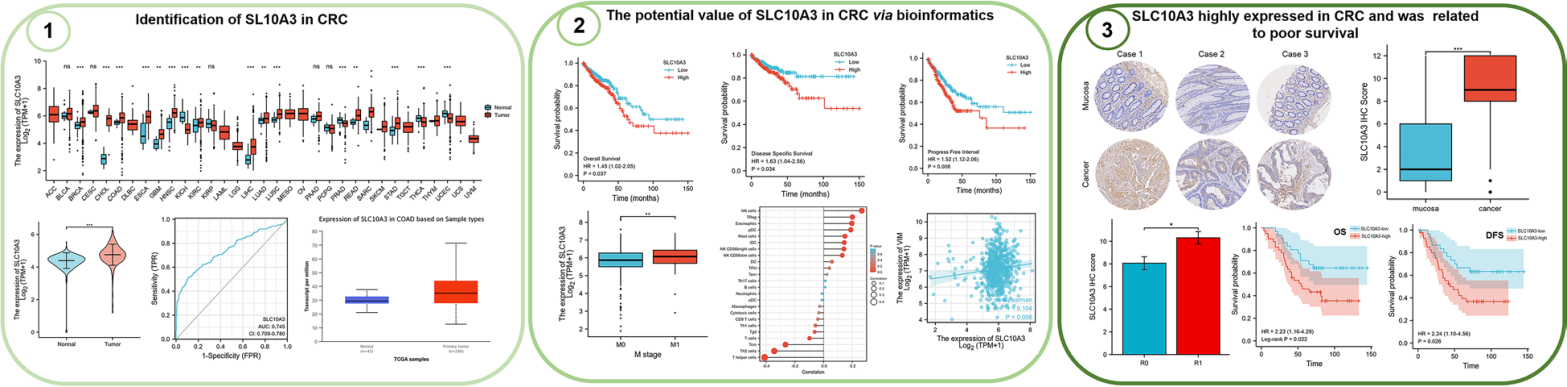

**Supplementary Information:**

The online version contains supplementary material available at 10.1186/s40001-023-01526-4.

## Introduction

Colon cancer is a major human cancer, ranking the top three cancers in the United States in terms of incidence as well as mortality in 2022 [1]. According to the data from 2014, it accounts for about 10% of all cancer cases in China [2, 3]. Colorectal cancer (CRC), including colon and rectum cancer, is often grouped due to their common characteristics, primarily manifesting as adenocarcinomas [4]. CRC can result from a combination of age, lifestyle, and genetic factors [5]. The progression from polyps to CRC is multifactorial, involving gene mutations, epigenetic modifications, and local inflammatory changes. Over the past 30 years, extensive research has identified numerous molecular alterations that contribute to the development and advancement of colon polyps. This research has unveiled multiple pathways leading to the heterogeneous presentation of colon polyps and CRC, characterized by global DNA abnormalities, including aneuploidy and microsatellite instability, as well as specific patterns of epigenetic changes (e.g., CpG Island Methylator Phenotype or CIMP), mutations in specific genes, and aberrantly methylated genes [6]. Comprehensive transcriptomics confer a precise characterization of individual cancers that should help to improve clinical strategies for neoplastic diseases through the development of novel drugs. In the past decade, numerous gene expression studies on CRC have been reported, aided by advanced technologies such as microarray and RNA-sequencing, as well as public databases such as Gene Expression Omnibus (GEO) and The Cancer Genome Atlas (TCGA). For instance, Liang, Li, and Zhao employed GEO datasets to identify a total of 3500 differentially expressed genes (DEGs) in CRC, consisting of 1370 upregulated and 2130 downregulated genes [7]. Pathway enrichment analysis revealed that upregulated DEGs were enriched in the cell cycle and DNA replication, while downregulated DEGs were enriched in drug metabolism. Additionally, Y. Guo, Bao, Ma, and Yang aimed to identify potential key candidate genes and pathways in CRC using four GEO cohorts and discovered 292 shared DEGs (165 upregulated and 127 downregulated) from the four datasets [8]. In another study (Wu, Wu, & Jiang, 2017) [9], gene and microRNA (miRNA) expression profiles from GEO datasets were utilized to identify 600 upregulated DEGs, 283 downregulated DEGs, 13 upregulated miRNAs, and 7 downregulated miRNAs [10].

The Solute Carrier (SLC) Family 10 (SLC10) is involved in the influx transport of bile acids, steroidal hormones, specific drugs, and a variety of other substrates, and comprises seven family members, including SLC10A1, SLC10A2, SLC10A3, SLC10A4, SLC10A5, SLC10A6 and SLC10A7. The SLC10 family is identified as the Bile Acid Sodium Symporter (BASS) family in the transporter classification system and belongs to the bile/arsenite/riboflavin transporter (BART) superfamily [11]. Bile acids (BA) are complex signaling molecules that modulate glucose, lipid and energy metabolism and are also implicated in the etiology of certain types of cancer [11]. Recent studies suggest that SLC10A3 may influence chemotherapy resistance and serve as a promising immune biomarker in liver cancer. Moreover, the expression of SLC10A3 protein is correlated with stromal CD4 T cells, CD20 B cells, macrophages, PDCD1 (PD-1) and CD274 (PD-L1) expression, indicating a potential regulatory role in liver cancer immunotherapy [12]. However, whether SLC10A3 plays a similar role in colorectal adenocarcinoma remains unknown.

Currently, updated public databases based on integrative bioinformatics analysis of the Cancer Genome Atlas (TCGA) have significantly enhanced the efficiency of identification of biomarkers and functional genes in cancerous diseases. Therefore, this study evaluates the transcriptional profiles and potential prognostic value of the SLC10A3 by systematical bioinformatics analysis and provides a novel role of SLC10A3 in the prognostic value and immune microenvironment in colorectal adenocarcinoma.

## Material and methods

### Transcriptome data sources

The mRNA expression data of SLC10A3 were downloaded from the TCGA database (http://cancergenome.nih.gov/abouttcga) for 33 different types of cancer [13]. The transcriptome data were then transformed into Log2 form and analyzed using the Wilcoxon rank-sum test to compare the expression levels between tumor and normal tissues. A *p*-value < 0.05 was considered statistically significant. To evaluate the predictive value of SLC10A3 for COAD and READ, AUC and ROC analysis were conducted.

### Survival analysis

The hazard ratio (HR) and 95% confidence intervals were calculated using univariate survival analysis. To assess the survival outcomes of SLC10A3 in COAD and READ, Kaplan–Meier survival analysis was conducted for OS (overall survival), DSS (disease-specific survival), and PFI (progression-free interval). The survival differences were compared using ‘‘survminer’’ and ‘‘surviva’’ packages. The concordance index (C-index) was used to evaluate the discrimination of the nomograms.

To explore the DNA methylation of SLC10A3 in TCGA, MethSurv7 was utilized. The analysis involved assessing the methylation levels and prognostic values of each CpG site within SLC10A3. Furthermore, the relationship between the CpG methylation status of SLC10A3 and the overall survival (OS) of CRC was evaluated.

### Analysis of mutation

For the genetic alteration analysis of SLC10A3, we utilized the cBioPortal (https://www.cbioportal.org/), a comprehensive website offering visualization, analysis, and download options for large-scale cancer genomics datasets [14]. Specifically, we examined the alteration frequency, mutation type, and copy number alterations (CNA) of SLC10A3 in COAD and READ.

### Immune cells infiltration

We utilized ssGSEA [15] to investigate the relationship between SLC10A3 and 24 types of immune cell infiltration in the immune microenvironment of CRC. Furthermore, we examined the differences in immune cell abundance between the high and low SLC10A3 expression groups. Using the Tumor Immune Estimation Resource (TIMER) [16], we assessed the association between the expression levels of tumor-infiltrating immune cells, including B cells, CD4 + T cells, CD8 + T cells, neutrophils, macrophages, dendritic cells, and cancer-associated fibroblasts (CAFs), and SLC10A3 expression. The Pearson method was used to display the results. Finally, we explored the correlation between somatic copy number alterations (SCNA) of SLC10A3 and the abundance of 6 leukocytes using the ‘SCNA module’.

### Functional enrichment analysis

We employed the Cluster Profiler R package to perform Gene Ontology (GO) and Kyoto Encyclopedia of Genes and Genomes (KEGG) pathway enrichment analyses of the DEGs between the high and low SLC10A3 expression groups.

### Immunohistochemical (IHC) staining

To assess the expression of SLC10A3 in human CRC tissues, we obtained the ZL-COC sur1602 CRC tissue microarrays from Shanghai Zhuolibiotech Company Co., Ltd. (Shanghai, China). Immunohistochemistry (IHC) was performed directly on the tissue microarrays, orderly dewaxed in xylene baths for 3 times, then rehydrated with graded alcohol series and retrieved in a pressure cooker with sodium citrate buffer (pH 6.0) heating for 15 min. The SLC10A3 (Proteintech, 19909-1-AP) antibody was utilized for IHC according to their protocols. The IHC steps followed our previous research [17]. We took two field shots on each slide. The IHC staining scores (IS) were classified into four score ranks: 0, negative; 1, weak; 2, moderate; and 3, strong. The percentage of positively stained cells (PS): 0 (< 5%), 1 (5–25%), 2 (25–50%), 3 (50–75%) and 4 (75–100%). The score of each slide: IS x PS (0–12) [18]. Additionally, clinical and pathological information regarding the tissue samples was obtained from the array manufacturer. Based on the cut-off value (*n* = 7), we classified those with > 7 as the SLC10A3-high group and those with ≤ 7 as the SLC10A3-low group.

### Statistical analysis

We performed log2 transformation to normalize all data obtained from TCGA. Spearman's or Pearson's test was used to analyze the correlation between two variables. TCGA (https://portal.gdc.cancer.gov/) COAD and READ data were utilized for these analyses. For survival analysis, we calculated the hazard ratios (HRs) and *p*-value using either the univariate Cox regression analysis or log-rank test. A *p*-value < 0.05 was considered statistically significant for all analyses.

## Results

### High expression of SLC10A3 in colorectal cancer samples

**Fig. 1:**
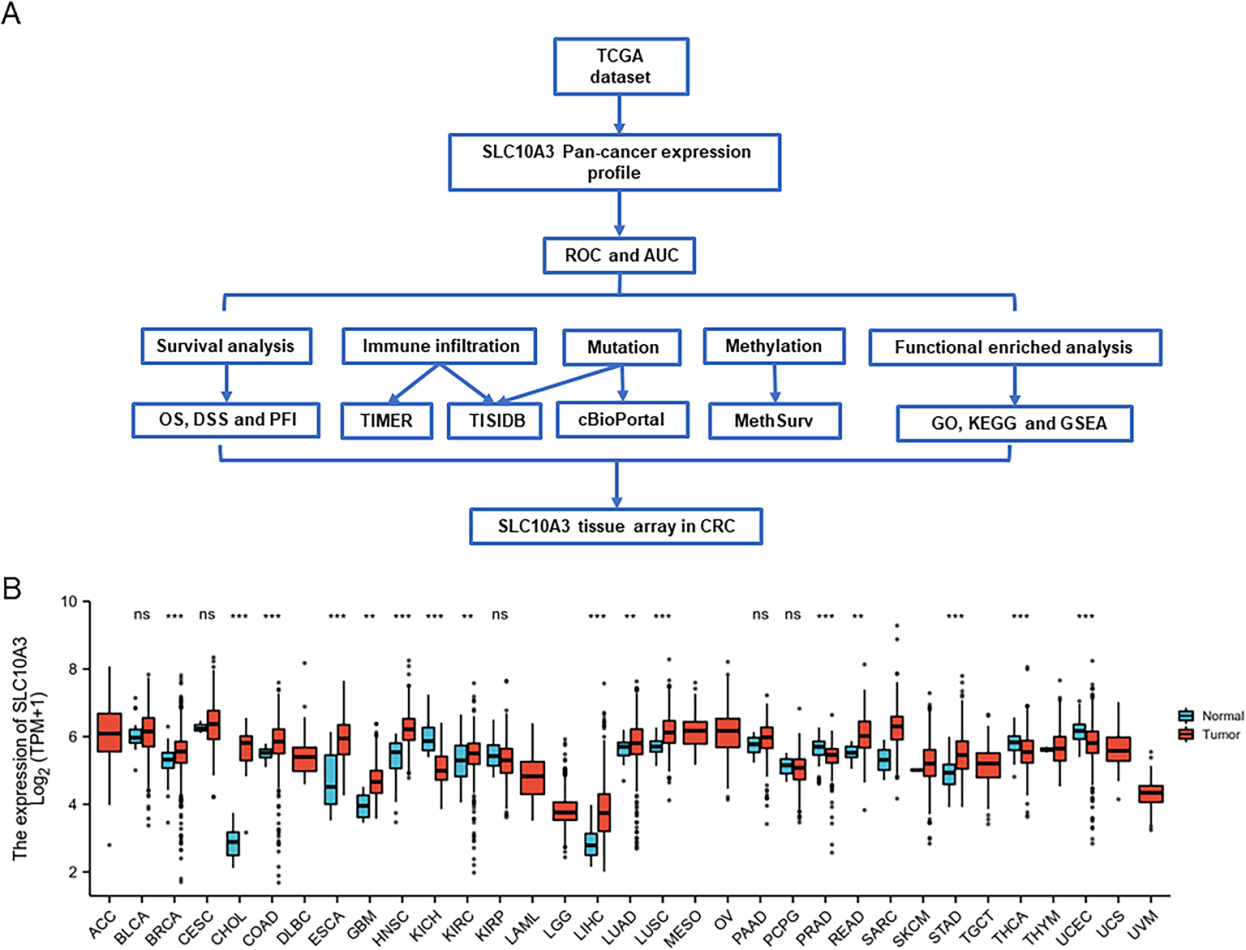
Identify SLC10A3 as a novel biomarker in colorectal cancer. **A** Flowchart of the present research. **B** SLC10A3 expression in different tumor and tumor-adjacent tissues in the TCGA database. * indicates *p*-value < 0.05; ** indicates *p*-value < 0.01; *** indicates *p*-value < 0.001

**Fig. 2:**
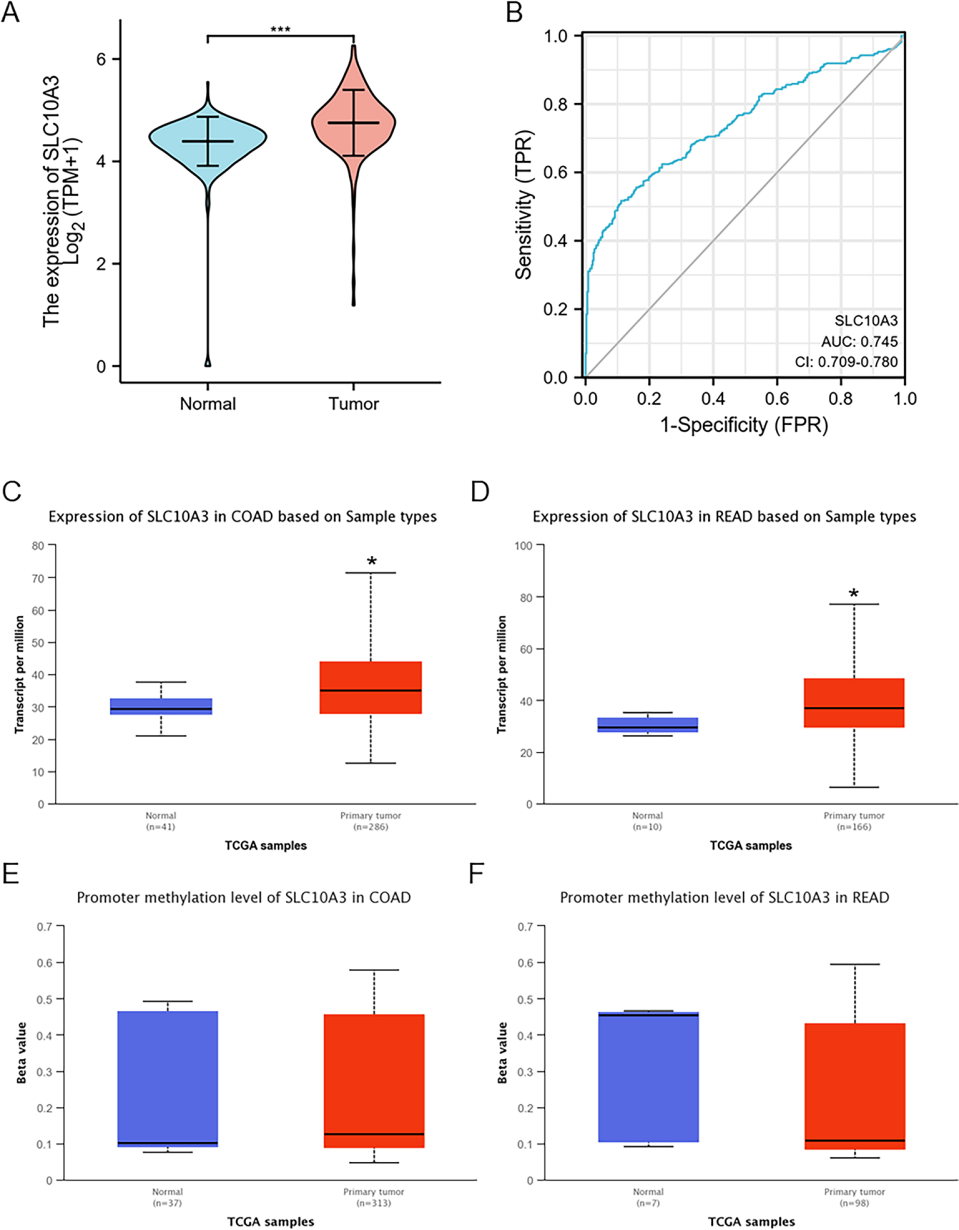
Comparative analysis of SLC10A3 expression and methylation levels in colorectal cancer (CRC) and normal tissues based on the TCGA database. **A** SLC10A3 expression was higher in CRC than in normal tissues based on the TCGA database. **B** The ROC and AUC of CRC is based on the expression of SLC10A3. **C**–**D** SLC10A3 expression was higher in COAD and READ than in normal tissues based on the TCGA database. **E**–**F** the methylation levels of SLC10A3 in COAD, READ and normal tissues. *indicates *p*-value < 0.05; ** indicates *p*-value < 0.01; *** indicates *p*-value < 0.001. *CRC* colorectal cancer, *READ* rectal adenocarcinoma; *COAD* colon adenocarcinoma

To explore the biological function of SLC10A3 in pan-cancer, especially CRC, we performed a series of bioinformatics analyses (Fig. 1A). Firstly, we compared the transcriptional levels of SLC10A3 in pan-cancer between tumor and normal samples by applying the TCGA databases. In TCGA data, differential SLC10A3 expression was significant among 16 of the 33 cancer types analyzed, including 12 upregulation and 4 downregulation (Fig. 1B). Moreover, SLC10A3 expression was higher in tumors than in normal tissues in CRC (Fig. 2A). We evaluated the accuracy of a colon cancer risk prediction model based on SLC10A3 using ROC curve analysis and found that the sensitivity was greater than the specificity, with an area under curve (AUC) of 0.745 (Fig. 2B). This indicates good predictive accuracy for the model. Based on the TCGA database and UALCAN, we found SLC10A3 was highly expressed in COAD and READ (Fig. 2CD). Based on the TCGA database, we found the methylation levels of SLC10A3 did not significantly change in colon and rectum cancer tissues than that in the corresponding normal tissues (Fig. 2EF).

### Mutation of SLC10A3 in CRC

**Fig. 3:**
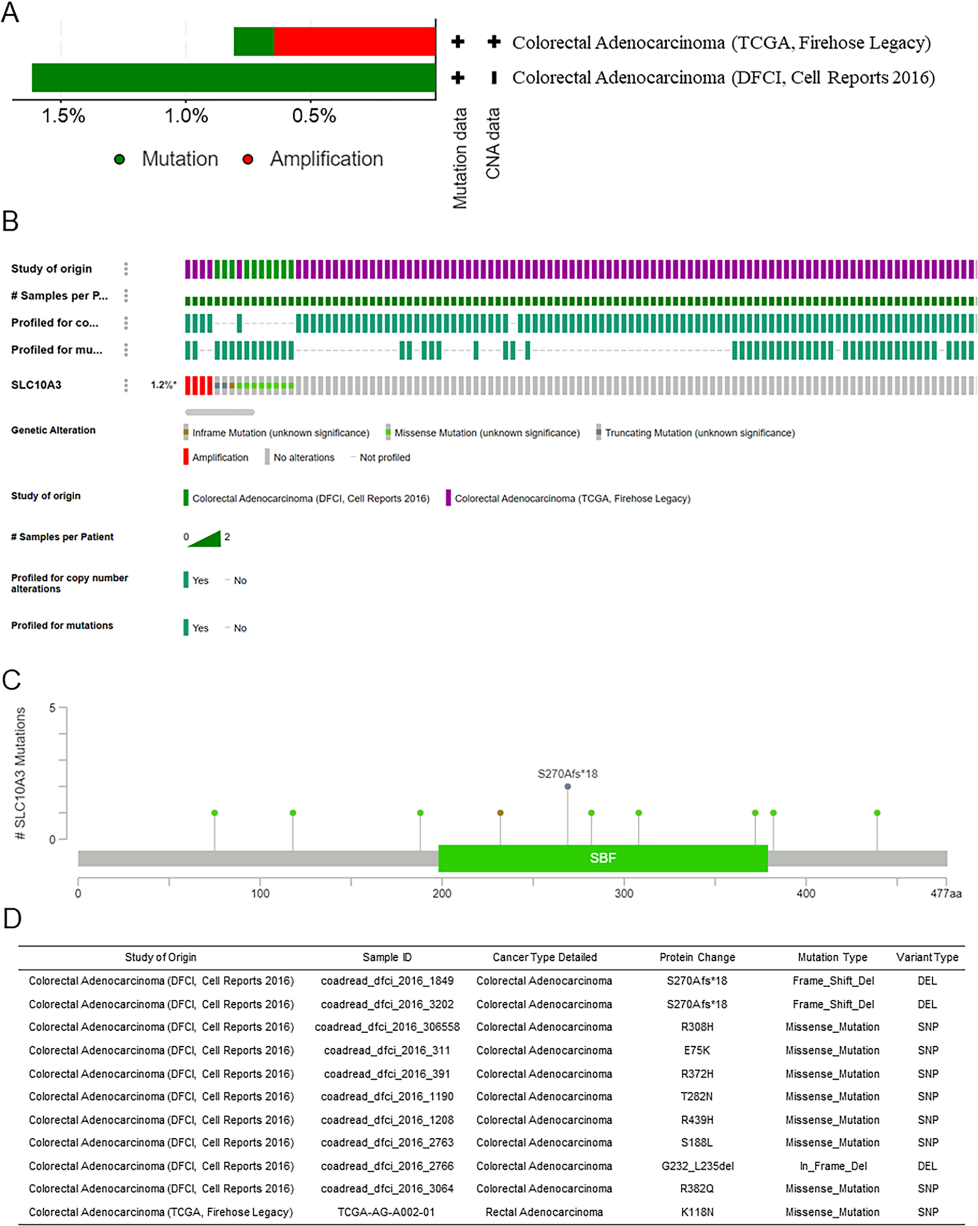
The genetic alterations of SLC10A3. **A** Alterations summary of SLC10A3 in TCGA CRC datasets. **B**–**D** Summary of SLC10A3 structural variant, mutations, and copy-number alterations. *CRC* colorectal cancer

Gene mutations play a crucial role in the development and progression of cancer. To gain insights into the mutations associated with CRC, we conducted a study on SLC10A3 gene mutations. Our analysis of the cBioPortal database revealed that SLC10A3 gene alterations were detected in CRC patients with amplification (> 0.5%) (Fig. 3A). The genetic alterations in SLC10A3 were primarily categorized into four types: amplification, truncating mutation, inframe mutation, and missense mutation. We further investigated the types, sites, and case numbers of SLC10A3 gene modification using data from DFCI, Cell Reports 2016, and TCGA Firehose Legacy (Fig. 3B–D).

### Association of SLC10A3 with clinical stage and prognosis

**Fig. 4:**
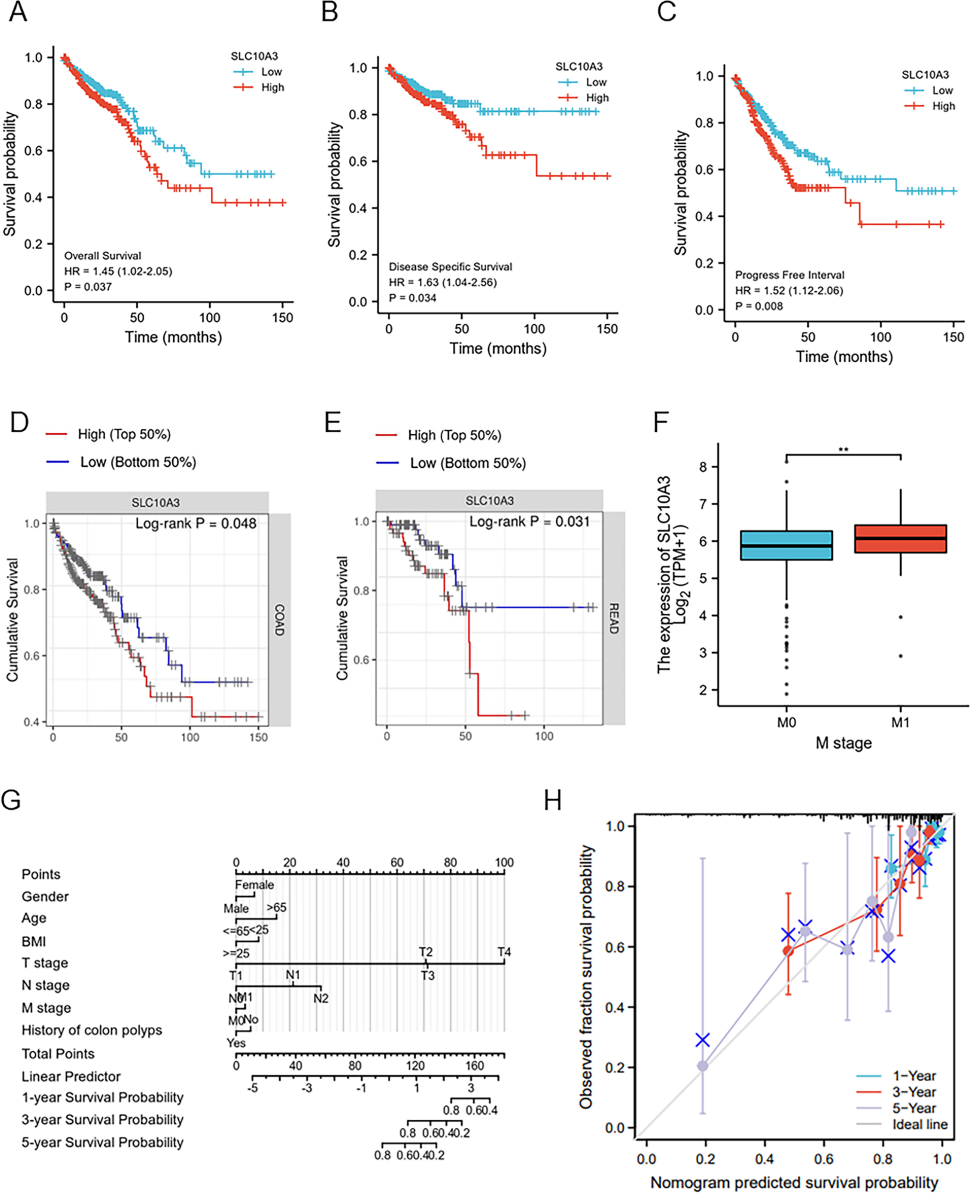
High-expressional SLC10A3 affects the prognosis of CRC patients. **A** The OS of CRC patients between low and high-expressional groups. **B** The DSS of CRC patients between low and high-expressional groups. **C** The PFI of CRC patients between low and high-expressional groups. **D**–**E** The survival analysis of SLC10A3 high expression and low expression in COAD and READ based on the TIMER database. **F** SLC10A3 expression in CRC patients was analyzed based on the tumor metastasis. **G**–**H** Nomogram to predict the COAD survival possibility among 1 year, 3 years and 5 years, including age, gender, TNM stage, tumor stage, and risk score. CRC, colorectal cancer; READ, rectal adenocarcinoma; COAD, colon adenocarcinoma. *HR* hazard ratio, *OS* overall survival, *DSS* disease-special survival, *PFI* progress-free interval

We examined the association between SLC10A3 expression and clinical stage in CRC, using high and low expression groups. Our analysis revealed positive correlations between SLC10A3 and lymph node invasion (*p*-value = 0.006), distant metastasis (*p*-value < 0.001), and patient age (*p*-value = 0.049) (Table 1). Moreover, we found that the mRNA level of SLC10A3 was higher in M1 stage than in M0 stage (Fig. 4F). In order to investigate the relationship between SLC10A3 expression and patient survival, we employed the Kaplan–Meier (KM) Plotter. Our results demonstrated that higher mRNA levels of SLC10A3 were significantly associated with shorter OS (*p*-value = 0.037), DSS (*p*-value = 0.034), and PFI (*p*-value = 0.008) in COAD patients (Fig. 4A–C). However, we also observed that high expression of SLC10A3 was linked to shorter OS (*p*-value = 0.022) and DFS (p-value = 0.026) (Fig. 4D–E). To assess survival rates in COAD patients, we constructed a nomogram based on age, gender, TNM stage, tumor stage, and risk score, and found good consistency between predicted and actual survival rates (Fig. 4G–H).

**Table 1:** Clinicopathological characteristics of the patient cohorts

Characteristic	Low expression of SLC10A3	High expression of SLC10A3	P-value
n	322	322	
T stage, n (%)			0.817
T1	11 (1.7%)	9 (1.4%)	
T2	58 (9%)	53 (8.3%)	
T3	216 (33.7%)	220 (34.3%)	
T4	34 (5.3%)	40 (6.2%)	
N stage, n (%)			0.006
N0	203 (31.7%)	165 (25.8%)	
N1	63 (9.8%)	90 (14.1%)	
N2	53 (8.3%)	66 (10.3%)	
M stage, n (%)			< 0.001
M0	249 (44.1%)	226 (40.1%)	
M1	29 (5.1%)	60 (10.6%)	
Age, median (IQR)	69 (59, 77)	66 (57, 75)	0.049

To confirm the prognostic significance of SLC10A3 in CRC patients, we conducted univariate and multivariate survival analyses (Tables 2, 3). Univariate analysis identified four prognostic factors in CRC: age (≤ 65 vs. > 65), N stage (N1–2 vs. N0), M stage (M1 vs. M0), and T stage (T1 vs. T4). Further multivariate analysis revealed that age (*p*-value = 0.019), BMI (*p*-value = 0.035), and N stage (p*-v*alue < 0.001) were independent predictors of OS in CRC patients (Table 2).

**Table 2:** Univariate and multivariate analyses of SLC10A3 in colorectal adenocarcinoma

Parameters	Univariate analysis				Multivariate analysis		
	Number	HR	95%CI	*p*-value	HR	95%CI	*p*-value
Gender	643			0.769	1.377	0.769–2.464	0.282
Male	342	Ref					
Female	301	0.949	0.671–1.344	0.769			
Age	643			< 0.001***	2.076	1.125–3.828	0.019*
< = 65	276	Ref					
> 65	367	1.939	1.320–2.849	< 0.001***			
BMI	329			0.09	0.55	0.316–0.959	0.035*
< 25	107	Ref					
> = 25	222	0.649	0.394–1.069	0.09			
T.stage	640			< 0.001***	12,132,270.68	0.000−Inf	0.996
T1	20	Ref			3.825	1.847–7.921	< 0.001***
T2	111	1	0.216–4.639	1			
T3	435	2.047	0.504–8.317	0.316			
T4	74	6.151	1.458–25.953	0.013*			
N.stage	639			< 0.001***	3.825	1.847–7.921	< 0.001***
N0	367	Ref					
N1	153	1.774	1.131–2.781	0.013*			
N2	119	3.873	2.588–5.796	< 0.001***			
M.stage	563			< 0.001***	1.4	0.667–2.937	0.373
M0	474	Ref					
M1	89	3.989	2.684–5.929	< 0.001***

**Table 3:** The relationship of SLC10A3 and immune cell infiltration in colorectal adenocarcinoma

Gene	Immune cell	Cor_pearson	*P* value_pearson
SLC10A3	aDC	0.119308778	0.002367691
SLC10A3	B cells	0.087328833	0.026333164
SLC10A3	CD8 T cells	0.296041467	1.49E−14
SLC10A3	Cytotoxic cells	0.067264383	0.087343934
SLC10A3	DC	0.14491996	0.000216769
SLC10A3	Eosinophils	0.334674996	2.13E−18
SLC10A3	iDC	0.280039956	4.02E−13
SLC10A3	Macrophages	− 0.096063281	0.01450874
SLC10A3	Mast cells	0.230838999	2.84E−09
SLC10A3	Neutrophils	− 0.033541229	0.394352235
SLC10A3	NK CD56bright cells	0.385570409	2.32E−24
SLC10A3	NK CD56dim cells	0.233923458	1.72E−09
SLC10A3	NK cells	0.525750235	2.89E−47
SLC10A3	pDC	0.192697675	7.88E−07
SLC10A3	T cells	− 0.027027185	0.492546183
SLC10A3	T helper cells	− 0.391843934	3.57E-25
SLC10A3	Tcm	− 0.458856982	5.25E−35
SLC10A3	Tem	− 0.060462111	0.124450625
SLC10A3	TFH	0.189667921	1.18E−06
SLC10A3	Tgd	0.037105772	0.346024191
SLC10A3	Th1 cells	− 0.045568799	0.247085786
SLC10A3	Th17 cells	0.119111108	0.002407874
SLC10A3	Th2 cells	− 0.276374634	8.30E−13
SLC10A3	TReg	0.30131108	4.80E−15

### Role of SLC10A3 in CRC immune microenvironment

**Fig. 5:**
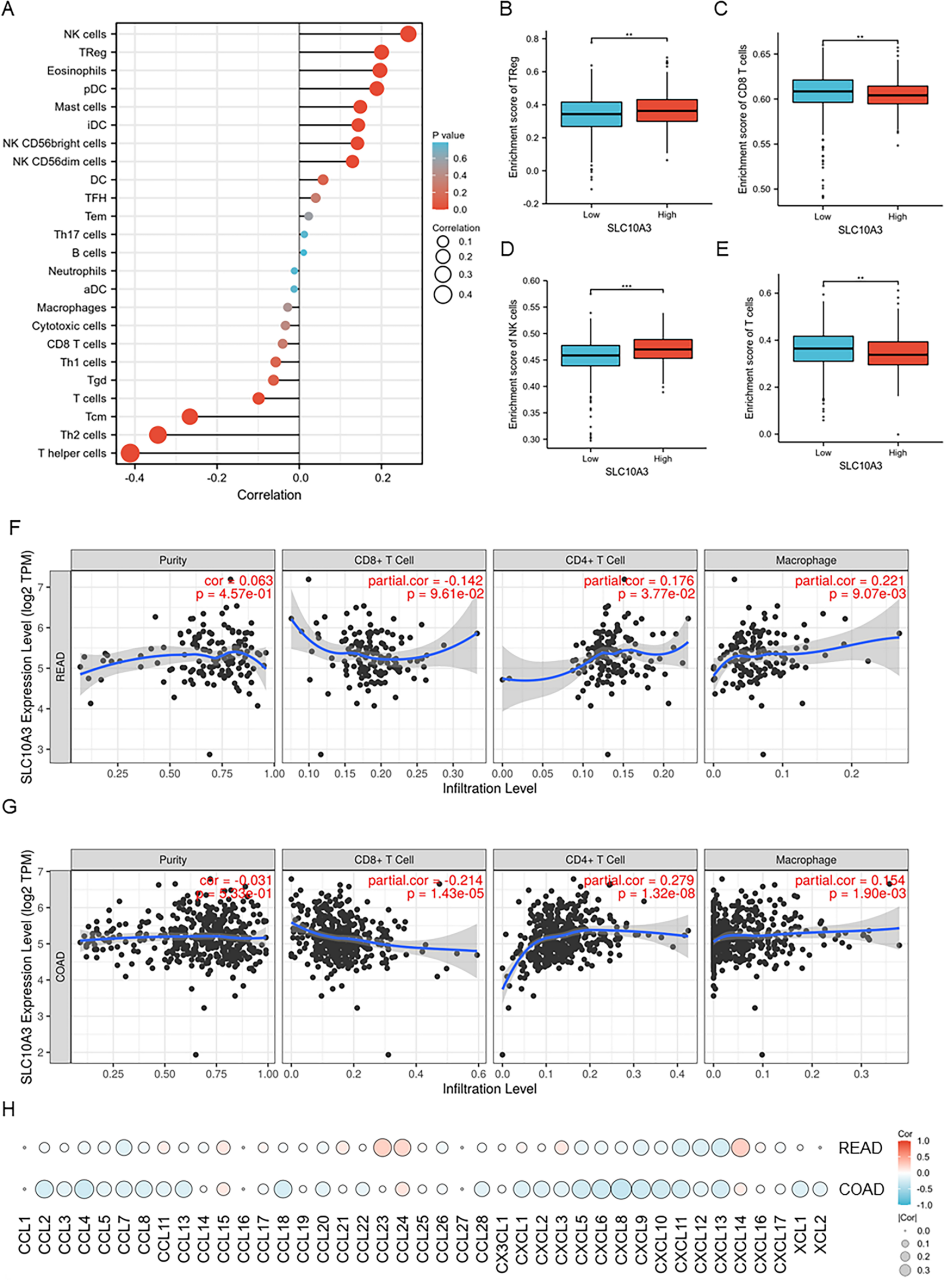
Correlation of immune cell infiltration, chemokines and SLC10A3 expression in CRC patients. **A** Relationships among infiltration levels of 24 immune cell types and SLC10A3 expression profiles by Spearman’s analysis in CRC. **B**–**E** The infiltration levels of Treg cells, CD8 + cells, NK cells, and T cells in the high- and low-SLC10A3 expression groups in CRC. **F**–**G** SLC10A3 expression is related to immune infiltration levels in COAD and READ through TIMER. **H** The relationship between chemokines and SLC10A3 expression in COAD and READ. *CRC* colorectal cancer; *READ* rectal adenocarcinoma, *COAD* colon adenocarcinoma. *p-value < 0.05; **p-value < 0.01; ***p-value < 0.001

The tumor microenvironment plays a crucial role in tumor genesis and development. Therefore, investigating the relationship between SLC10A3 and immune cell infiltration is significant. Immune cell infiltration in CRC was determined using ssGSEA for 24 immune cell types, and the relationship between SLC10A3 expression and immune cell infiltration was analyzed using Pearson's correlation coefficient (Table 3). NK cells, Eosinophils, and TReg showed a positive correlation with SLC10A3 expression, while Tcm, Th2 cells, and T helper cells showed a negative association with SLC10A3 (Fig. 5A). The tumor infiltration levels of TReg cells, CD8 + cells, NK cells, and T cells were consistent with the Pearson's correlation analysis results (Fig. 5B–E). Furthermore, we analyzed the relationship between somatic copy number aberrations (CNA) and tumor immune infiltration using TIMER. Firstly, the ‘‘SCNA’’ module analysis showed that altered SLC10A3 gene copy numbers in COAD were associated with immune cell infiltration levels of B cells, macrophages, neutrophils, and dendritic cells (Fig. S1). In comparison, SLC10A3 gene copy numbers were only associated with dendritic cell infiltration levels in READ (Fig. S1). Secondly, the ‘‘Gene’’ module analysis confirmed that immune infiltration levels of B cells, CD8 + T cells, CD4 + T cells and macrophages were associated with SLC10A3 expression in READ and COAD (Fig. 5FG). Besides, the results of CIBERSORT, EPIC, QUANTISEQ, XCELL, and MCPCOUNTER about the relationship between SLC10A3 and immune infiltration are displayed in Table S1. In conclusion, high expression of SLC10A3 could affect the immune microenvironment of READ and COAD by regulating the proportions of immune cell infiltration.

Chemokine networks affect tumor immunity and tumorigenesis by regulating the tumor microenvironment. Therefore, we next examined the correlations between chemokines and SLC10A3 in CRC. The results showed significant (p-value < 0.05) and strong positive correlations between SLC10A3 expression and the following chemokines: CCL15, CCL24, and CXCL14. Additionally, strong negative correlations were observed between SLC10A3 expression and chemokines including CCL2-5/7–8, CXCL5-6 and CXCL8-13 in COAD (Fig. 5H). These results further confirmed that SLC10A3 was significantly correlated with immune infiltrating cells and chemokines in READ and COAD, indicating that SLC10A3 may play a crucial role in the microenvironment of READ and COAD.

### Correlation analysis of SLC10A3 and cancer-associated fibroblasts

**Fig. 6:**
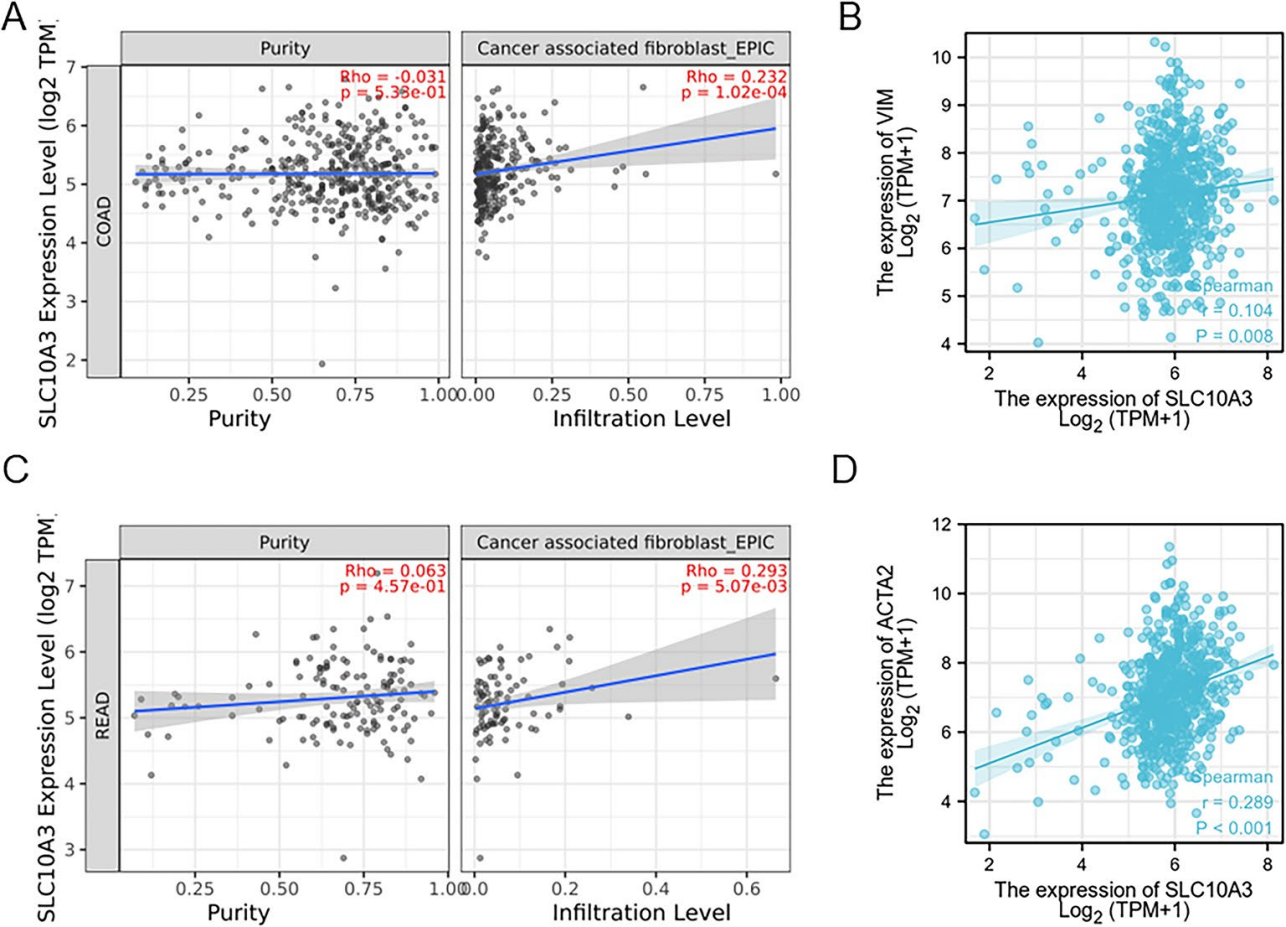
Correlation analysis of SLC10A3 and cancer-associated fibroblasts in COAD and READ. **A**, **C** The association of SLC10A3 and cancer-associated fibroblast in COAD (*p*-value = 1.02e−04) and READ (*p*-value = 5.07e-03) by EPIC. **B**, **D** The correlation analysis between the SLC10A3 expression and the expression levels of VIM, ACAT2 in the TCGA dataset. *READ* rectal adenocarcinoma, *COAD* colon adenocarcinoma

Cancer-associated fibroblasts (CAFs) create a conducive environment for tumor growth, as they are known to inhibit immune cell function through secretion of cytokines and metabolites, thereby promoting tumorigenesis, invasion, metastasis, and drug resistance. Our analysis of the correlation between SLC10A3 and CAFs using TIMER revealed a significant association in CRC (Fig. 6AC). Specifically, high expression of SLC10A3 was positively correlated with CAF markers such as ACAT2 and VIM in COAD and READ (Fig. 6BD).

### Association of SLC10A3 and DNA mismatch repair (MMR)

**Fig. 7:**
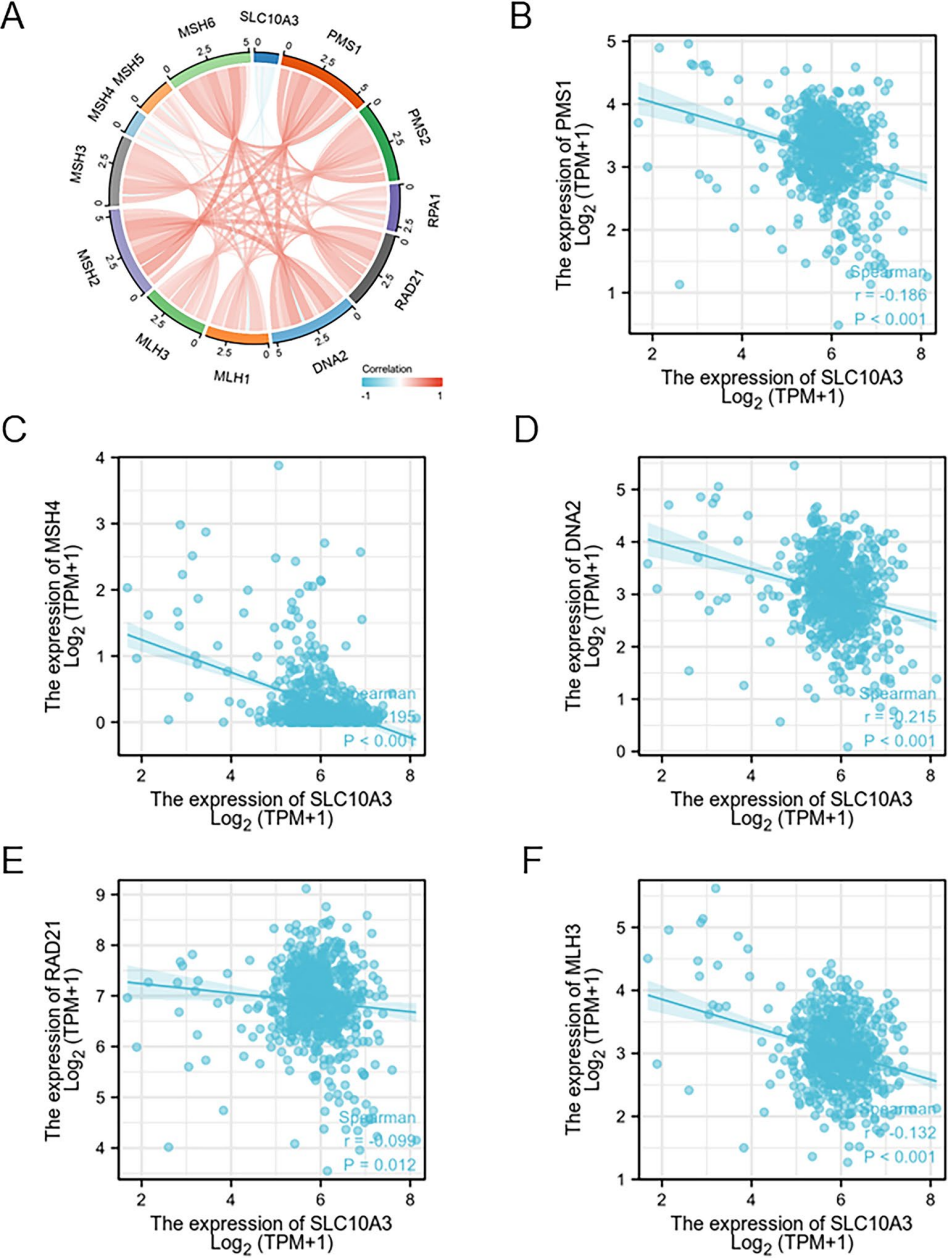
Correlation analysis between SLC10A3 and immune checkpoint factors in CRC. **A**–**F** The correlation analysis between the SLC10A3 expression and the expression levels of PMS1, RAD21, DNA2, MLH3, MLH1, PMS2, MSH2, MSH6 and MSH4 in the TCGA-CRC dataset

We analyzed the correlations between SLC10A3 expression and mismatch repair molecules using Spearman’s correlation coefficient. Obviously, SCL10A3 expression showed a negative association with PMS1, RAD21, DNA2, MLH3, and MSH4 (Fig. 7A–F). In summary, our findings suggest a negative link between SLC10A3 and MMR in CRC, with implications for error repair of nucleotide sequence and microsatellite instability.

### The methylation status of the SLC10A3 is associated with the prognosis of COAD and READ patients

DNA methylation levels in SLC10A3 were analyzed, and the prognostic value of CpG islands in SLC10A3 was assessed using the MetSurv tool. The findings revealed the presence of 10 methylated CpG islands in COAD and 11 methylated CpG islands in READ. Notably, methylation levels of two specific CpG islands, namely cg06266461 and cg09418475, showed significant association with prognosis (*p*-value < 0.05) in COAD. In contrast, no CpG islands in READ were found to be associated with prognosis (Table 4).

**Table 4:** The prognostic value of SLC10A3 CpG islands

Name	Cancer	HR	CI	*P*-value
cg03025340	COAD	1.485	(0.81;2.722)	0.20067
cg03210912	COAD	0.659	(0.407;1.068)	0.090432
cg05424879	COAD	0.654	(0.404;1.06)	0.084865
cg06266461	COAD	0.601	(0.368;0.982)	0.042236
cg06616857	COAD	0.657	(0.406;1.063)	0.086871
cg08648877	COAD	0.687	(0.421;1.12)	0.132196
cg09418475	COAD	0.604	(0.372;0.981)	0.041632
cg11233153	COAD	0.697	(0.428;1.138)	0.148766
cg11667509	COAD	0.628	(0.387;1.02)	0.060052
cg21109542	COAD	0.733	(0.454;1.183)	0.203073
cg23493704	COAD	1.284	(0.714;2.312)	0.404056
cg24546622	COAD	0.643	(0.397;1.042)	0.073
cg03025340	READ	0.675	(0.255;1.788)	0.428583
cg03210912	READ	2.755	(0.968;7.841)	0.057553
cg05424879	READ	1.5	(0.566;3.974)	0.414421
cg06266461	READ	1.541	(0.44;5.395)	0.499002
cg06616857	READ	2.146	(0.486;9.479)	0.313706
cg08648877	READ	1.519	(0.435;5.305)	0.512058
cg09418475	READ	1.751	(0.638;4.809)	0.276973
cg11233153	READ	1.444	(0.554;3.758)	0.452065
cg11667509	READ	1.484	(0.422;5.219)	0.538587
cg21109542	READ	1.45	(0.548;3.84)	0.454143
cg23493704	READ	0.708	(0.245;2.045)	0.523718
cg24546622	READ	1.579	(0.573;4.356)	0.377332

### Screening DEGs and functional enrichment analyses based on SLC10A3 single gene analysis

**Fig. 8:**
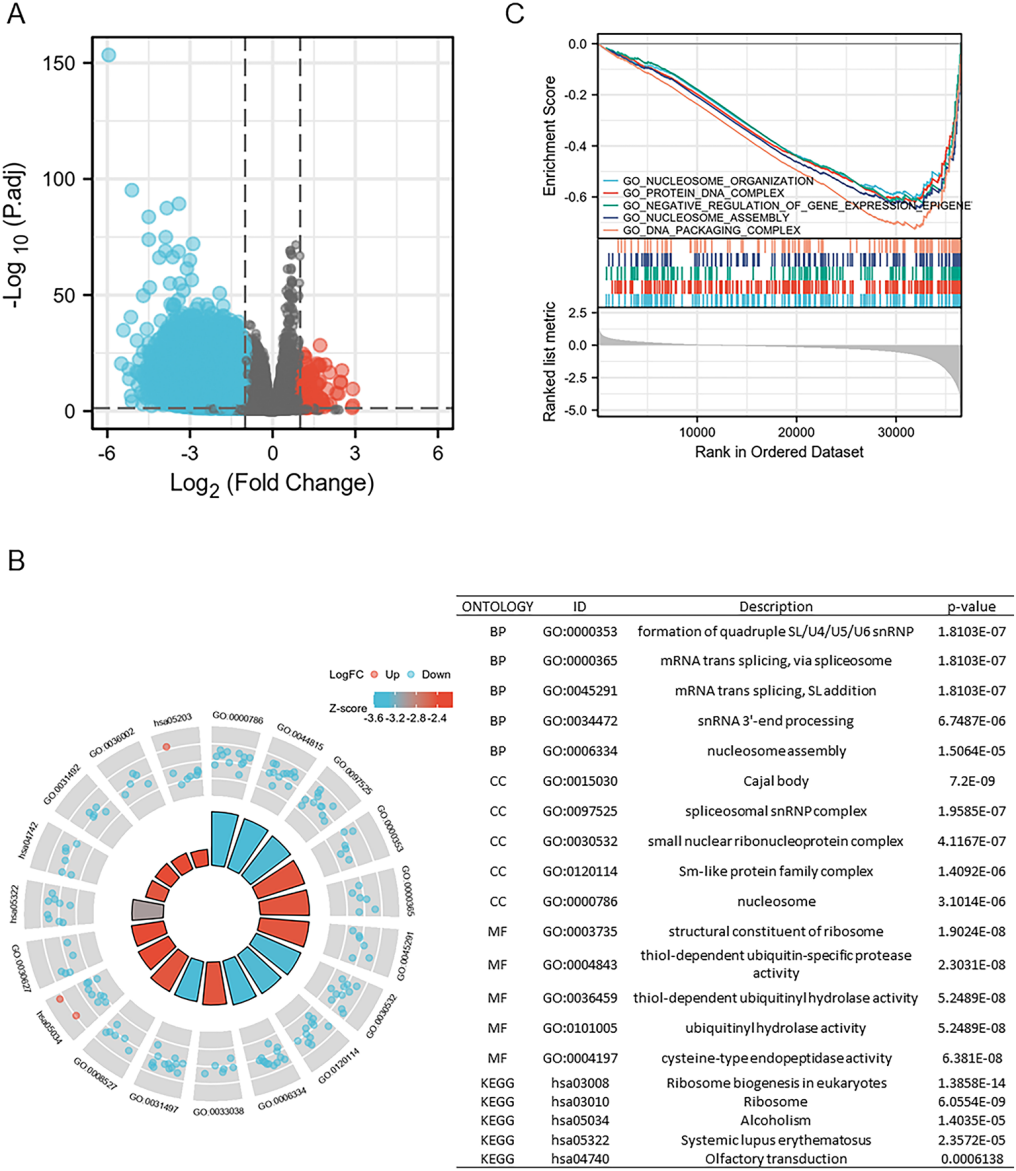
Screening DEGs and constructing functional analysis between SLC10A3 high-expressional group and SLC10A3 low-expressional group. **A** Volcano plots of DEGs between the expression of SLC10A3-high and SLC10A3-low in CRC samples. **B** GO and KEGG analysis among DEGs in CRC as a circle graph. **C** GSEA analysis of DEGs between SLC10A3-high and SLC10A3-low in CRC samples. *CRC* colorectal cancer

Based on the TCGA database, we conducted SLC10A3 single gene analysis, divided the tumor samples into SLC10A3 low expression group and SLC10A3 high expression group by R (version 4.1.1), and screened differentially expressed genes in volcano plot (Fig. 8A). DEGs, including 127 upregulated and 2492 downregulated genes, were identified between low and high SLC10A3 groups in CRC (Fig. 8A). The potential bio-function of DEGs was enriched in nucleosome, nucleosome assembly and small nuclear ribonucleoprotein complex via GO and KEGG enrichment analysis in CRC (Fig. 8B). SLC10A3 may promote CRC progression by increasing chromatin synthesis. GSEA showed that the SLC10A3-associated DEGs were significantly enriched in nucleosome organization, protein–DNA complex and nucleosome assembly to regulate the progression of CRC (Fig. 8C).

### High SLC10A3 expression is strongly associated with CRC prognosis

**Fig. 9:**
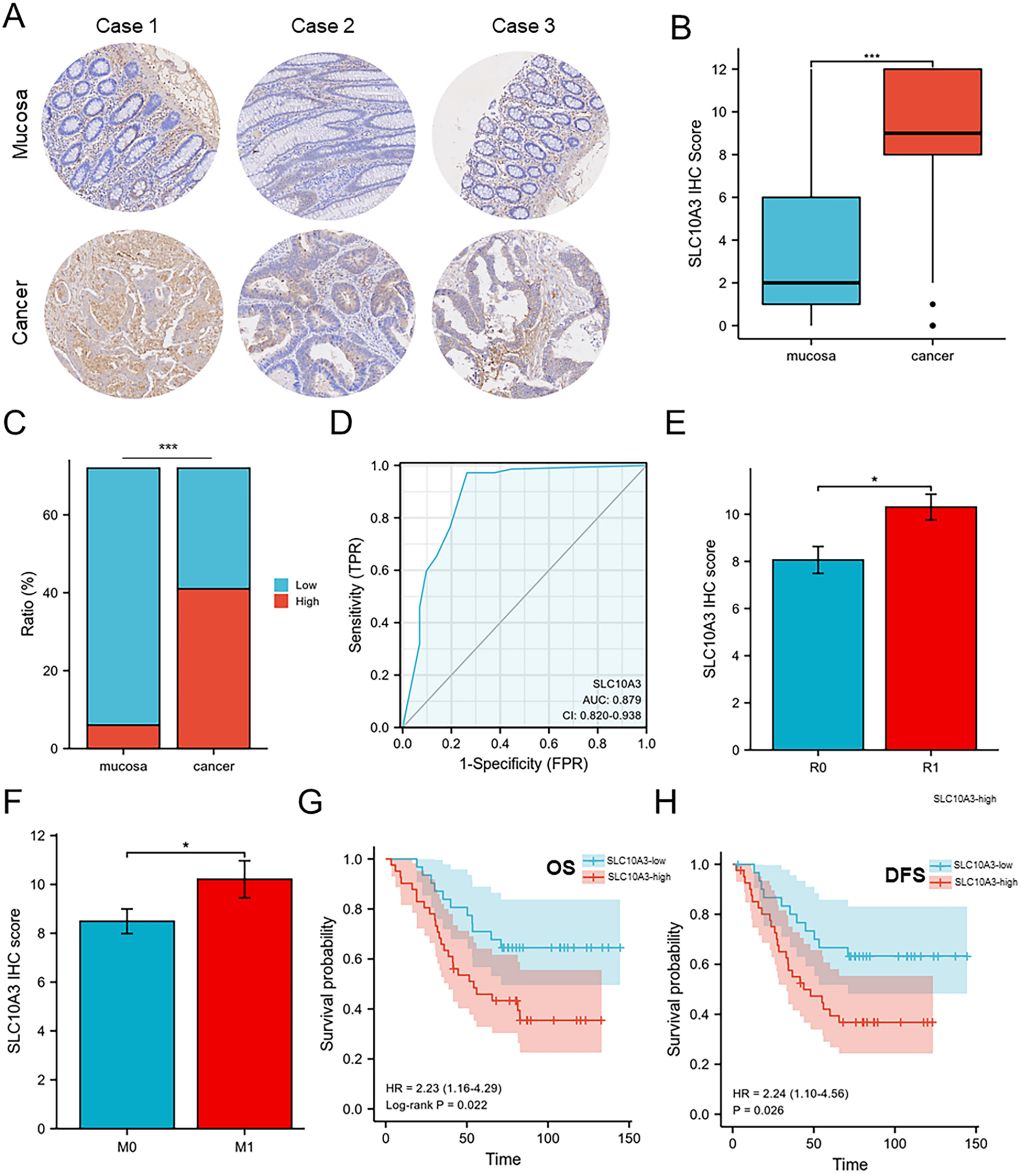
High expression of SLC10A3 predicts poor prognosis in CRC patients. **A** IHC analysis of SLC10A3 expression in a CRC patient tissue array (*n* = 72). SLC10A3 levels in cancer tissues and corresponding normal tissues. Representative images are shown. Scale bars = 100 μm. **B** IHC scores of SLC10A3 in human CRC tissues and adjacent tissues. **C** The percent of high and low SLC10A3 expression in CRC patients (normal and cancer tissues). **D** ROC curve with corresponding AUC value for SLC10A3 when classifying cancer from the mucosa. **E**–**F** SLC10A3 expression was positively associated with tumor relapse (R) and metastasis (M). **G**–**H** High SLC10A3 expression predicted low OS (HR = 2.23, *p*-value = 0.022) and DFS (HR = 2.24, *p*-value = 0.026). Data were shown as mean and standard deviation from three independent experiments. Data are means ± SDs. *, *p*-value < 0.05; **, *p*-value < 0.01; ***, *p*-value < 0.001. *CRC* colorectal cancer

We investigated the functions of SLC10A3 in CRC by examining the relative levels of SLC10A3 in patient tissues and corresponding normal tissues. 72 patient specimens were analyzed, and SLC10A3 was found to be expressed differently, with some specimens having low levels and others having high levels (Fig. 9A–B). Using IHC scores (cut-off = 7), we divided the specimens into high and low expression groups, with a higher proportion of high SLC10A3 expression in tumor tissues (Fig. 9C). Additionally, the amount of SLC10A3 was significantly increased in CRC tissues compared to normal tissues (Fig. 9C). To predict the diagnostic value of SLC10A3 in distinguishing CRC from the mucosa, we used ROC and AUC, which resulted in an AUC value of 0.879 (Fig. 9D). Our analysis indicated that the level of SLC10A3 is correlated with CRC malignancy in tumor relapse and metastasis (Fig. 9E–F), but not in lymph node invasion status and tumor stage (Fig. S2AB). Besides, high SLC10A3 expression predicted low OS (HR = 2.23, *p*-value = 0.022) and DFS (HR = 2.24, *p*-value = 0.026) (Fig. 9G–H). Our data suggest that SLC10A3 may serve as a useful diagnostic and prognostic indicator in CRC patients.

## Discussion

Colorectal cancer is the third most common cancer worldwide in terms of incidence and ranks fourth in terms of cancer-related mortality [19]. Although significant clinical advancements have been made in the treatment of colorectal cancer through medical techniques, surgical interventions, and chemotherapy [20], the current lack of an effective diagnostic method to monitor CRC progression or recurrence remains a challenge. Recent studies have highlighted the pivotal role of SLC10A3 in various cancers, with its mutation being implicated in malignant growth and cancer development. Specifically, SLC10A3 plays a critical role in regulating the immune response in liver cancer, related with stromal CD4 T cells, CD20 B cells, and macrophage [9]. Upregulation of SLC10A3 mRNA is statistically related with unfavorable survival outcomes and immune infiltration among low-grade gliomas [21]. Nonetheless, there is currently no literature on SLC10A3 in colorectal cancer. Thus, this study aims to elucidate the expression of SLC10A3, immunocorrelation and its significant role in predicting the prognosis of colorectal cancer, ultimately establishing a genetic risk-scoring model.

In our study, through TCGA database we found that SLC10A3 is significantly higher expressed in colorectal cancer, so we further investigated the role of SLC10A3 in colorectal cancer and unveiled its prognostic value, functional enrichment pathways, methylation patterns, tumor immune infiltration, and variance analysis between high and low SLC10A3 expression groups using bioinformatic analysis. Higher SLC10A3 expression was notably associated with worse prognosis, including shorter overall survival (OS, *p*-value = 0.037, HR = 1.45(1.02–2.05)), disease-free survival (DFS, *p*-value = 0.034, HR = 1.63(1.04–2.56)), and progression-free survival (PFI, *p*-value = 0.008, HR = 1.52(1.12–2.06)). Moreover, high SLC10A3 expression may be linked to metastasis in CRC patients. Abnormal methylation of specific genes in cells poses a high risk of cancer, and this aberrant methylation occurs during cell oncogenesis [22, 23]. The methylation levels of four CpG islands, including cg06266461 and cg09418475, were found to be associated with prognosis (*p* < 0.05) in COAD. Tissue microarray of CRC confirmed the high expression of SLC10A3 in tumor tissues, which was indicative of poor prognosis, including OS (*p*-value = 0.022, HR = 2.23(1.16–4.29) and DFS (*p*-value = 0.026, HR = 2.24(1.10–4.56)). Our results demonstrate that CRC patients with high SLC10A3 expression experienced shorter survival times and a higher likelihood of disease progression, highlighting SLC10A3 as an independent risk factor for survival.

Mounting evidence indicates the pivotal role of the immune microenvironment in tumorigenesis [24]. Tumor immunity closely intertwines with cell growth, and any disruption in the immune microenvironment can expedite cancer progression [25]. Computer algorithms analyzing transcriptomic data have gained widespread adoption in assessing the dynamic immune components within the tumor microenvironment (TME) of cancer patients. In this study, we employed the ssGSEA method to determine the extent of immune cell infiltration and the ratio of immune/mesenchymal components in colorectal cancer (CRC) samples sourced from the TCGA database. Our findings confirmed the prognostic biomarker, SLC10A3, as an influential factor in immune regulation within the CRC TME. CD8 + T cell can effectively kill various pathogens and tumor cell, while Treg cell plays a crucial role in inhibitory tumor immune microenvironment. These findings affirmed the substantial correlation between SLC10A3 and CD8 + T cell (negatively) and Treg cell (positively) in CRC, suggesting a crucial role of SLC10A3 within the microenvironment of these malignancies.

Subsequently, we conducted correlation analyses between chemokines and SLC10A3 in CRC. Spearman's method was utilized to examine the associations between SLC10A3 expression and immune checkpoint molecules. Additionally, we investigated the relationships between SLC10A3 and various chemokines in both COAD and READ. The results revealed statistically significant (*p*-value < 0.05) and robust positive correlations with the several chemokines: CCL15, CCL24, and CXCL14. Moreover, strong negative correlations were observed between SLC10A3 and chemokines such as CCL2-5/7–8, CXCL5-6 and CXCL8-13 in COAD. Cancer-associated fibroblasts (CAFs) represent a critical component within the tumor microenvironment, exerting a pivotal role in tumor initiation and progression [26, 27]. Rather than existing in isolation, CAFs interact with neighboring tumor cells, actively promoting their growth, survival, and sustaining their malignant behavior [28]. By secreting diverse cytokines, CAFs establish communication with other stromal cells and tumor cells, resulting in immune cell function inhibition and facilitation of tumor development [29]. Cytokines such as TGF-β are secreted by CAFs, leading to DC maturation suppression and Treg differentiation promotion [30–32]. Consequently, we assessed the correlation between SLC10A3 and CAFs in COAD and READ by utilizing R and TIMER, based on TCGA datasets. Notably, we identified a significant positive correlation between SLC10A3 and CAFs in both COAD (p-value = 1.02e−04) and READ (*p*-value = 5.07e−03). High expression levels of SLC10A3 were found to be positively associated with CAF markers, including ACAT2 (p-value = 0.008) and VIM (*p*-value < 0.001).

The mismatch repair system is a safety net in the body that maintains the integrity and stability of genetic material. In DNA mismatch repair (MMR), repair genes can ensure the ‘‘fidelity’’ of the replication process during DNA replication [33]. Our results showed SCL10A3 expression was negatively associated with PMS1 (*p*-value < 0.001), RAD21 (*p*-value = 0.012), DNA2 (*p*-value < 0.001), MLH3 (*p*-value < 0.001), and MSH4 (*p*-value < 0.001). Low expression in mismatch repair genes can lead to shorten or lengthen microsatellite segments. Microsatellite instability (MSI) will result in a high mutant phenotype of the genome, leading to an increased risk of tumor development [34].

However, it is important to acknowledge the limitations of our study. Our analysis heavily relied on tissue samples from the TCGA dataset, with only 72 pairs of clinical samples used for validation. Therefore, it would be beneficial to validate our findings using larger sample cohorts from different medical centers. Additionally, although we found an association between SLC10A3 and the immune microenvironment of colorectal cancer, confirmatory experiments were not conducted. Moving forward, our further plan includes verifying that SLC10A3 promotes CRC proliferation and metastasis through cytological and animal experiments, as well as exploring its mechanism. This aspect represents a potential future direction for our investigation.

## Conclusion

Overall, the findings suggested that SLC10A3 high expression is an independent risk biomarker for worse prognosis in patients with CRC and is related to immune infiltration and distant metastasis. Besides, our study may provide new insights into the treatment of CRC and inform further studies of tumor immunity in CRC. However, studies with larger clinical samples and further functional experiments are required to verify our results.

### Supplementary Information

**Additional file 1: Figure S1.** Correlation between somatic copy number alterations (SCNA) and immune infiltration abundance in relation to SLC10A3 expression. **Figure S2.** Impact of age, gender, and SLC10A3 expression on the overall and disease-free survival of CRC patients. **Table S1.** The relationship between SLC10A3 and immune infiltration based on TIMER2.

## Data Availability

The datasets generated and analyzed during this study are available in the public database TCGA.

## Abbreviations

SLC10: The solute carrier family 10
AUC: Area under the curve
ROC: Receiver operating characteristic
DEGs: Differentially expressed genes
GSEA: Gene set enrichment analysis
OS: Overall survival
DSS: Disease-specific survival
PFI: Progression-free interval
DFS: Disease-free survival
GEO: Gene expression omnibus
TCGA: The Cancer Genome Atlas
GO: Gene Ontology
KEGG: Kyoto Encyclopedia of Genes and Genomes
BASS: Bile acid sodium symporter
BART: Bile/arsenite/riboflavin transporter
BA: Bile acids
C-index: The concordance index
TIMER: Tumor immune estimation resource
IHC: Immunohistochemistry
IS: The IHC staining scores
PS: Positively stained cells
CNA: Copy number aberrations
MSI: Microsatellite instability
CRC: Colorectal cancer
READ: Rectal adenocarcinoma
COAD: Colon adenocarcinoma
TME: Tumor micro-environment
CAFs: Cancer-associated fibroblasts
SCNA: Somatic copy number alterations
HR: Hazard ratio
MMR: Mismatch repair
