# Supplementary material for: The impact of SLC10A3 on prognosis and immune microenvironment in colorectal adenocarcinoma
Source: Eur J Med Res. 2024 Jan 4;29:20. doi: 10.1186/s40001-023-01526-4 (PMC10765936; doi:10.1186/s40001-023-01526-4)
Supplement: Supplementary file 1 — Additional file 1: Figure S1. Correlation between somatic copy number alterations (SCNA) and immune infiltration abundance in relation to SLC10A3 expression. Figure S2. Impact of age, gender, and SLC10A3 expression on the overall and disease-free survival of CRC patients. Table S1. The relationship between SLC10A3 and immune infiltration based on TIMER2. [file 40001_2023_1526_MOESM1_ESM.docx]

**Additional file**

**The impact of SLC10A3 on prognosis and immune microenvironment in colorectal adenocarcinoma**

**Additional file**

**Figure S1. Correlation between somatic copy number alterations (SCNA) and immune infiltration abundance in relation to SLC10A3 expression.**

**
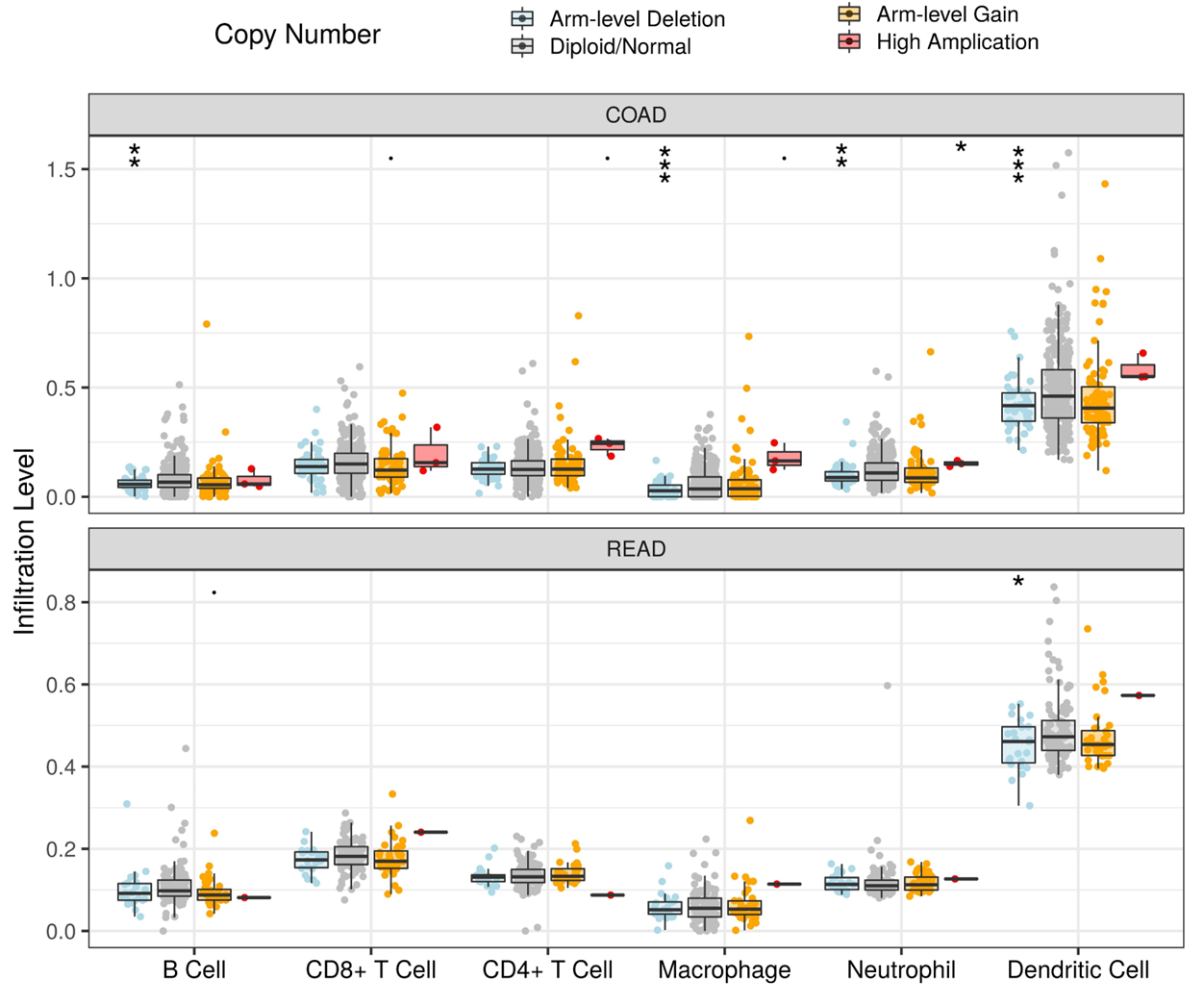
**

**Figure S2. Impact of age, gender, and SLC10A3 expression on the overall and disease-free survival of CRC patients.** (A-B) Female and older patients had shorter lifespans. (C-D) The OS, DFS of CRC patients between low and high-expressional groups. CRC, colorectal cancer; OS, overall Survival; DFS, disease-free survival.


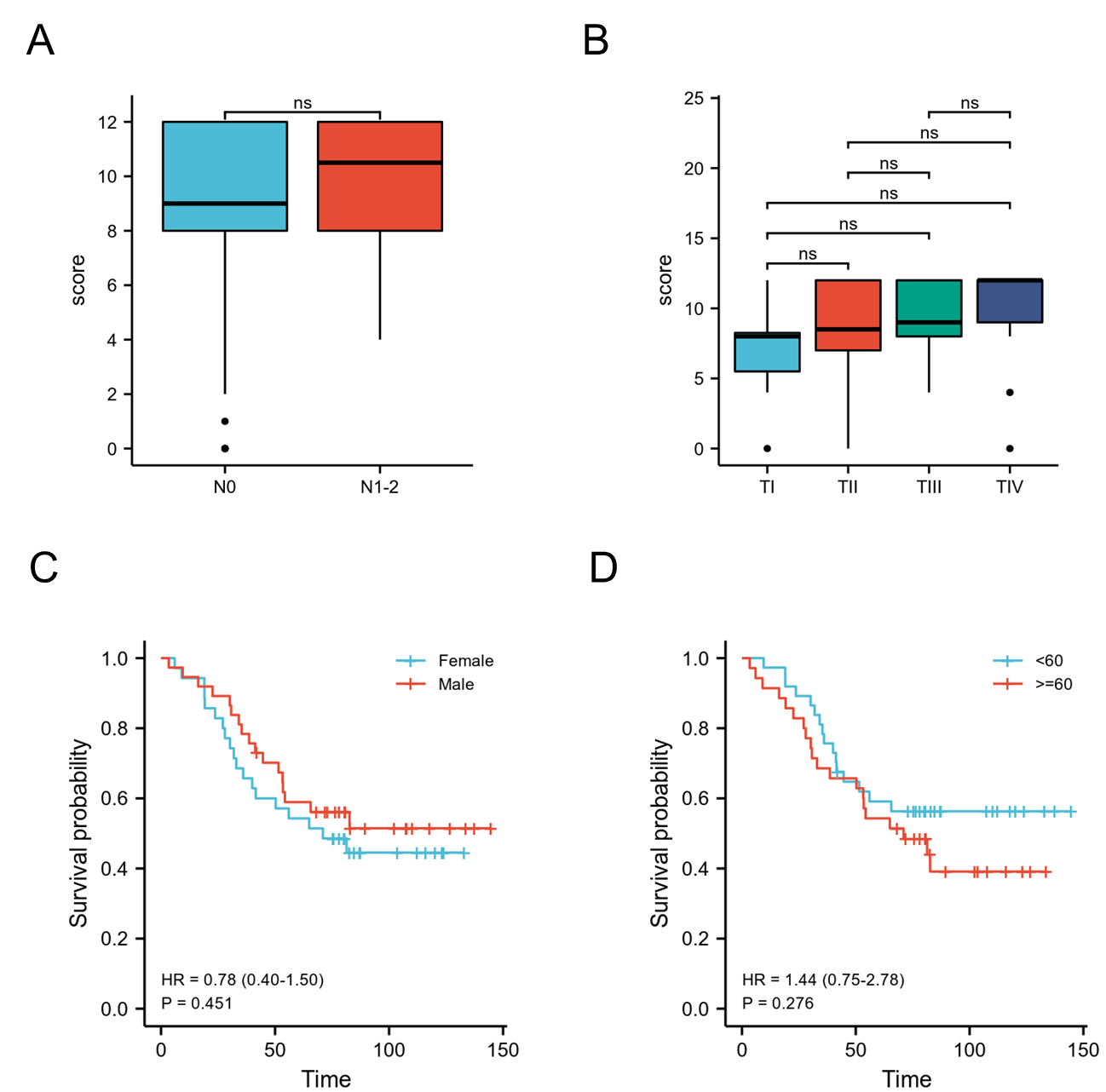


**Table S1. The relationship between SLC10A3 and immune infiltration based on TIMER2.**

|  |  |  |  |  |  |  |
| --- | --- | --- | --- | --- | --- | --- |
| cells | cancer | CIBERSORT | MCPCOUNTER | XCELL | EPIC | QUANTISEQ |
| B cell | COAD | NA | 0.116037447 | **-0.134340234** | 0.067802244 | 0.125860934 |
|  | READ | NA | -0.120133639 | -0.205546867 | -0.151622861 | -0.025159541 |
| T cell CD4+ | COAD | NA | NA | NA | -0.051902884 | NA |
|  | READ | NA | NA | NA | -0.067155024 | NA |
| T cell CD4+ Th1 | COAD | NA | NA | **-0.166997721** | NA | NA |
|  | READ | NA | NA | **-0.233949822** | NA | NA |
| T cell CD4+ Th2 | COAD | NA | NA | **-0.357168546** | NA | NA |
|  | READ | NA | NA | -0.091131431 | NA | NA |
| Endothelial cell | COAD | NA | **0.250330584** | **0.146631564** | **0.214756558** | NA |
|  | READ | NA | 0.096627109 | 0.017024299 | -0.022488518 | NA |
| T cell CD8+ | COAD | -0.061082013 | 0.014110805 | -0.064769103 | -0.105166106 | -0.00932946 |
|  | READ | -0.125408872 | -0.062486392 | -0.100179717 | -0.102499466 | -0.144052309 |
| Tregs | COAD | 0.113206373 | NA | 0.046410556 | NA | 0.00669494 |
|  | READ | -0.286205977 | NA | -0.00249584 | NA | -0.026556309 |
| DC | COAD | NA | 0.101842353 | -0.03216371 | NA | -0.006096826 |
|  | READ | NA | 0.126061717 | -0.003041335 | NA | 0.074144998 |
| NK cell | COAD | NA | **-0.179271029** | -0.101465105 | -0.022414074 | **0.175067733** |
|  | READ | NA | -0.139687185 | 0.203479813 | 0.140074475 | 0.144038468 |
| Mast cell | COAD | NA | NA | 0.026209618 | NA | NA |
|  | READ | NA | NA | -0.084602555 | NA | NA |
| Macrophage0 | COAD | **0.1506273** | NA | NA | NA | NA |
|  | READ | -0.015773217 | NA | NA | NA | NA |
| Macrophage 1 | COAD | -0.03403432 | NA | 0.037985599 | NA | **-0.222356313** |
|  | READ | 0.043471015 | NA | **0.238944569** | NA | 0.112419528 |
| Macrophage 2 | COAD | 0.051201597 | NA | 0.034437049 | NA | **0.239840824** |
|  | READ | **0.267123867** | NA | 0.149843875 | NA | 0.160963442 |
| Eosinophils | COAD | 0.080361799 | NA | 0.02564887 | NA | NA |
|  | READ | -0.04347 | NA | -0.063736959 | NA | NA |
| Neutrophil | COAD | -0.044647445 | **0.198156317** | 0.083288176 | NA | **0.146302967** |
|  | READ | 0.091712254 | 0.037301222 | -0.172140634 | NA | 0.179593922 |
| Cancer Associated Fibroblast | COAD | NA | **0.261849663** | **0.222652377** | **0.232172663** | NA |
|  | READ | NA | **0.293539062** | **0.186404694** | **0.2929987** | NA |

Blue negative relation, p-value<0.05; Red positive relation, p-value<0.05.
